# Prevalence and outcomes of patients with SMuRF-less acute coronary syndrome undergoing percutaneous coronary intervention

**DOI:** 10.1136/openhrt-2024-002733

**Published:** 2024-06-05

**Authors:** Jocasta Ball, Diem T Dinh, Angela Brennan, Andrew Ajani, David J Clark, Melanie Freeman, Ernesto Oqueli, Chin Hiew, Shane Nanayakkara, Antony Walton, James A Shaw, William Chan, Christopher M Reid, Dion Stub

**Affiliations:** 1 Centre of Cardiovascular Research and Education in Therapeutics, School of Public Health and Preventive Medicine, Monash University, Melbourne, Victoria, Australia; 2 Baker Heart and Diabetes Institute, Melbourne, Victoria, Australia; 3 Monash Alfred Baker Centre for Cardiovascular Research, Melbourne, Victoria, Australia; 4 Melbourne Private Hospital, Melbourne, Victoria, Australia; 5 Department of Cardiology, Austin Health, Heidelberg, Victoria, Australia; 6 Eastern Health, Box HIll, Victoria, Australia; 7 Grampians Health Ballarat, Ballarat, Victoria, Australia; 8 School of Medicine, Faculty of Health, Deakin University, Geelong, Victoria, Australia; 9 Geelong Hospital, Geelong, Victoria, Australia; 10 Department of Cardiology, Alfred Hospital, Melbourne, Victoria, Australia; 11 School of Translational Medicine, Monash University, Melbourne, Victoria, Australia; 12 School of Population Health, Curtin University, Perth, Western Australia, Australia

**Keywords:** Acute Coronary Syndrome, Percutaneous Coronary Intervention, RISK FACTORS

## Abstract

**Background:**

There is increasing awareness that patients without standard modifiable risk factors (SMuRFs; diabetes, hypercholesterolaemia, hypertension and smoking) may represent a unique subset of patients with acute coronary syndrome (ACS). We aimed to investigate the prevalence and outcomes of patients with SMuRF-less ACS undergoing percutaneous coronary intervention (PCI) compared with those with SMuRFs.

**Methods:**

We analysed data from the Melbourne Interventional Group PCI Registry. Patients with coronary artery disease were excluded. The primary outcome was 30-day mortality. Secondary outcomes included in-hospital and 30-day events. Long-term mortality was investigated using Cox-proportional hazards regression.

**Results:**

From 1 January 2005 to 31 December 2020, 2727/18 988 (14.4%) patients were SMuRF less, with the proportion increasing over time. Mean age was similar for patients with and without SMuRFs (63 years), and fewer females were SMuRF-less (19.8% vs 25.4%, p<0.001). SMuRF-less patients were more likely to present with cardiac arrest (6.6% vs 3.9%, p<0.001) and ST-elevation myocardial infarction (59.1% vs 50.8%, p<0.001) and were more likely to experience postprocedural cardiogenic shock (4.5% vs 3.6%, p=0.019) and arrhythmia (11.2% vs 9.9%, p=0.029). At 30 days, mortality, myocardial infarction, revascularisation and major adverse cardiac and cerebrovascular events did not differ between the groups. During median follow-up of 7 years, SMuRF-less patients had an adjusted 13% decreased rate of mortality (HR 0.87 (95% CI 0.78 to 0.97)).

**Conclusions:**

The proportion of SMuRF-less patients increased over time. Presentation was more often a devastating cardiac event compared with those with SMuRFs. No difference in 30-day outcomes was observed and SMuRF-less patients had lower hazard for long-term mortality.

**WHAT IS ALREADY KNOWN ON THIS TOPIC**

- Patients with acute coronary syndrome (ACS) without standard modifiable risk factors (SMuRFs) may represent a distinctive patient group. However, prior research on outcomes in this group is conflicting.

**WHAT THIS STUDY ADDS**

- In patients with ACS receiving percutaneous coronary intervention (PCI), SMuRF-less patients more frequently present with a catastrophic cardiac event. Conversely, short-term outcomes do not differ to those with SMuRFs and long-term outcomes favour SMuRF-less patients.

**HOW THIS STUDY MIGHT AFFECT RESEARCH, PRACTICE OR POLICY**

- Further research is needed to investigate the unique risk factors profile(s) of this patient group.

## Introduction

Despite major prevention and therapeutic advances, cardiovascular disease (CVD) remains the world’s leading cause of death. Within Australia, the total economic burden of CVD is projected to exceed US$140 billion in combined healthcare costs and productivity losses this decade.^1–3^ Acute coronary syndrome (ACS) accounts for 53% of all CVD admissions in Australia and 9 in every 10 dollars of CVD-related health expenditure.^4^

In ACS, prevention strategies have focused on standard modifiable risk factors (SMuRFs), defined as the presence of diabetes mellitus, hyperlipidaemia, hypertension and current smoking. However, there remains a group of patients without these risk factors (‘SMuRF-less’ patients) who develop disease despite not being identified as ‘at risk’. ACS occurs in approximately 30 million SMuRF-less individuals globally and is responsible for 1.4 million deaths per annum in this patient cohort.^5^ These individuals represent an increasing proportion of patients with ACS. In two cohorts of patients with ST-elevation myocardial infarction (STEMI), the proportion of SMuRF-less patients increased from 14% to 27% over one decade.^6 7^ Evidence suggests that SMuRF-less patients may paradoxically have an increased risk of mortality and poorer prognosis after an ACS event. One study found that patients with SMuRF-less STEMI had an approximately 50% increased risk of 30-day mortality than their counterparts with SMuRFs.^8^

SMuRF-less patients are often under-represented in clinical trials, and the burden, management and outcomes of these patients have not yet been fully elucidated. There is, therefore, a growing awareness of the importance of this underinvestigated group, which could raise several important questions for both clinicians and researchers. We, therefore, aimed to investigate the prevalence and outcomes of SMuRF-less patients compared with those with SMuRFs undergoing percutaneous coronary intervention (PCI) for ACS in Melbourne, Australia using a multicentre PCI registry.

## Methods

### Study design

We conducted a retrospective cohort study analysing data collected on consecutive PCI procedures included in the Melbourne Interventional Group (MIG) Registry between 1 January 2005 and 31 December 2020.

### Study population

Patients aged ≥18 years undergoing PCI for ACS were included. Patients without ACS and those for whom data for risk factors were not available were excluded from the analysis. Patients who experienced a previous MI (19.7%) or had a previous PCI/coronary artery bypass grafting (6.2%) were also excluded from the analysis.

### Data sources

The MIG registry, which has previously been described in detail,^9^ is a multicentre PCI registry that prospectively collects data on all PCI procedures at six tertiary referral hospitals in Victoria, Australia. Baseline, clinical and procedural characteristics are recorded on standardised case report forms at the time of index PCI, with 30-day outcome data collected by site nurse coordinators via telephone follow-up. An independent audit of several verifiable fields from 5% of enrolled patients is periodically conducted at all enrolling sites by an investigator not affiliated with the registry.^10^ Long-term mortality data are obtained via linkage between the registry and the National Death Index, an Australia-wide database of all patient deaths that is maintained by the Australian Institute of Health and Welfare.

### Study definitions

Presence or absence of SMuRFs was determined from medical records or patient self-report at the time of PCI by the clinician completing the MIG case report form. We used medical history and risk factor data collected at baseline and considered in-hospital diagnosis of hypertension, hyperlipidaemia or diabetes as equivalent to a prehospital diagnosis. Patients with these in-hospital diagnoses were, therefore, included in the SMuRF group for analysis. Indication for PCI was classified as STEMI, non-ST elevation MI (NSTEMI) and unstable angina (UA) presentations according to standard definitions.^11^ All in-hospital and 30-day outcomes were defined according to standard Academic Research Consortium definitions.^12^

### Study endpoints

The primary endpoint of this study was 30-day all-cause mortality. Secondary endpoints included in-hospital complications, 30-day outcomes and long-term mortality. In-hospital complications were recorded by clinicians during index PCI admission. Medication status and outcomes at 30 days, including all-cause mortality, recurrent acute myocardial infarction (AMI), stent thrombosis, major bleeding, target vessel revascularisation (TVR; repeat PCI or coronary artery bypass graft surgery), major adverse cardiovascular events (MACE; a composite of all-cause mortality, non-fatal AMI and TVR), major adverse cardiac and cerebrovascular events (MACCE; composite of all-cause mortality, non-fatal AMI, TVR and stroke) and readmission details were determined by patient telephone follow-up with confirmation of events using medical records.

### Patient and public involvement

Patients and the public were not involved in the design or conduct of our study, given the nature of the study as a retrospective analysis of existing, anonymised registry data. Patient/public involvement in the dissemination of study results will be sought.

### Statistical analysis

Categorical variables are presented as frequencies and proportions, and continuous variables are presented as median and IQR or mean and SD, where appropriate. Differences in categorical data between SMuRF-less patients and patients with ≥1 SMuRF were determined using the χ^2^ test. Differences in continuous data between the two groups were assessed using the independent t-test for normally distributed data and the Wilcoxon rank-sum test for non-parametric data.

Trend analysis was conducted using the nonparametric Cochran-Armitage test to understand whether there was a significant difference in the proportion of SMuRF-less patients over time during the study period. Multivariable logistic regression was conducted to investigate adjusted 30-day event rates between the two groups. Cox proportional hazards regression analysis was performed to identify unadjusted and adjusted HRs and 95% CIs for long-term mortality between the two groups. Potential confounders included in adjusted analyses were age (per year increase), sex, family history of coronary artery disease, cerebrovascular disease, peripheral vascular disease (PVD), estimated glomerular filtration rate (eGFR; ≥60, 30–59, <30 mL/min/1.73 m^2^), dialysis, chronic obstructive pulmonary disease (COPD), obstructive sleep apnoea, ACS presentation (STEMI, NSTEMI, UA), Killip class (1, ≥2), ejection fraction (>45%, 30%–45%, <30%), extent of coronary disease (single vessel, multivessel), use of a drug-eluting stent, out-of-hospital cardiac arrest (OHCA) on presentation, cardiogenic shock and arrhythmia. We also conducted stratified analyses to compare 30-day mortality between SMuRF-less patients and those with ≥1 SMuRF in males and females separately, in STEMI and NSTEMI only cohorts, and in those aged ≤65 years and those aged >65 years. Statistical analysis was conducted using Stata V.17.0 (Stata Corporation). A two-sided p value <0.05 was considered statistically significant.

## Results

### Baseline characteristics

Between 1 January 2005 and 31 December 2020, there were 18 988 individuals who underwent PCI for ACS, of whom 2727 were SMuRF-less (14.4%) and 16 261 had 1 or more SMuRFs. Trend analysis showed that there was a significant increase in the proportion of SMuRF-less patients over time from 11.1% in 2005 to 16.0% in 2020 (p <0.001) (online supplemental figure 1). Baseline characteristics of SMuRF-less patients compared with those with ≥1 SMuRF are presented in table 1. SMuRF-less patients were almost 1 year older than those with SMuRFs (63.4±12.5 years vs 62.6±12.4 years, respectively; p<0.001). In addition to the cohort including three times more males overall, there were also more males than females who were SMuRF-less (80.2% vs 74.6% of those with SMuRFs, p<0.001). SMuRF-less patients had a lower BMI compared with those with ≥1 SMuRF (27.3±4.8 vs 28.6±5.5), significantly more had normal renal function (eGFR ≥60 mL/min/1.73 m^2^; 83.5% vs 79.6%), and a lower presence of comorbidities including cerebrovascular disease (1.2% vs 4.2%), PVD (0.6% vs 3.0%), COPD (3.2% vs 5.2%) and obstructive sleep apnoea (2.0% vs 3.3%).

10.1136/openhrt-2024-002733.supp1 Supplementary data

**Table 1:** Baseline characteristics of the cohort stratified by the presence or absence of standard modifiable risk factors (SMuRFs)

Characteristic	Category	Total n=18 988	SMuRF-less n=2727	≥1 SMuRF n=16 261	P value
Age (years), mean (SD)		62.7 (12.4)	63.4 (12.5)	62.6 (12.4)	<0.001
Gender, n (%)	Male	14 316 (75.4%)	2187 (80.2%)	12 129 (74.6%)	<0.001
	Female	4671 (24.6%)	540 (19.8%)	4131 (25.4%)	
BMI (kg/m^2^), mean (SD)		28.4 (5.4)	27.3 (4.8)	28.6 (5.5)	<0.001
Cerebrovascular disease, n (%)		721 (3.8%)	33 (1.2%)	688 (4.2%)	<0.001
Peripheral vascular disease, n (%)		508 (2.7%)	17 (0.6%)	491 (3.0%)	<0.001
Rheumatoid arthritis, n (%)		324 (1.8%)	56 (2.1%)	268 (1.7%)	0.160
Family history of CAD, n (%)		6604 (37.3%)	852 (33.2%)	5752 (38.0%)	<0.001
eGFR (mL/min/1.73 m^2^), n (%)	≥60	14 370 (80.2%)	2113 (83.5%)	12 257 (79.6%)	<0.001
	30 to <60	3217 (17.9%)	403 (15.9%)	2814 (18.3%)	
	<30	341 (1.9%)	15 (0.6%)	326 (2.1%)	
Dialysis, n (%)		110 (0.6%)	4 (0.1%)	106 (0.7%)	0.001
Rhythm, n (%)	AF	759 (4.7%)	105 (4.5%)	654 (4.7%)	0.890
	SR	15 057 (93.2%)	2175 (93.4%)	12 882 (93.2%)	
	Other	340 (2.1%)	48 (2.1%)	292 (2.1%)	
Prior chronic heart failure, n (%)		679 (3.6%)	81 (3.0%)	598 (3.7%)	0.065
Previous valvular surgery, n (%)		69 (0.4%)	10 (0.4%)	59 (0.4%)	0.980
Chronic obstructive pulmonary disease, n (%)		936 (4.9%)	87 (3.2%)	849 (5.2%)	<0.001
Asthma, n (%)		1012 (5.3%)	151 (5.5%)	861 (5.3%)	0.600
Obstructive sleep apnoea, n (%)		589 (3.1%)	54 (2.0%)	535 (3.3%)	<0.001

AF, atrial fibrillation; BMI, body mass index; CAD, coronary artery disease; eGFR, estimated glomerular filtration rate; SD, standard deviation; SR, sinus rhythm.

### Clinical presentation

Table 2 shows the clinical presentation at the time of PCI of SMuRF-less patients compared with those with ≥1 SMuRF. Significantly, more SMuRF-less patients presented with OHCA (6.6% vs 3.9% of those with ≥1 SMuRF, p<0.001). Almost 8.5% more SMuRF-less patients presented with STEMI than those with ≥1 SMuRF (59.1% vs 50.8%, p<0.001). A greater proportion of SMuRF-less patients had a Killip Class of 1 and NYHA Class I compared with those with SMuRFs (76.6% vs 73.9% and 75.7% versus 68.4%, respectively; both p<0.001) but a lesser proportion had an ejection fraction>45% (73.3% vs 76.7%, p<0.001). Compared with their counterparts with SMuRFs, SMuRF-less patients were more likely to present with single vessel disease (57.1% vs 51.1%, p<0.001) and with the left anterior descending artery as the culprit vessel (46.2% vs 39.3%, p<0.001). An ostial lesion was present in a greater proportion of SMuRF-less patients (9.0% vs 7.2%, p=0.001) as was a Thrombolysis in Myocardial Infarction (TIMI) flow pre-PCI of 0 (43.2% vs 35.2%, p<0.001). There was no difference in TIMI flow post-PCI or percutaneous entry location between the patient groups. A greater proportion of SMuRF-less patients experienced transient no reflow (4.2% vs 3.1%, p—0.012).

**Table 2:** Clinical presentation at the time of PCI stratified by the presence or absence of SMuRFs

Characteristic	Category	Total n=18 988	SMuRF-less n=2727	≥1 SMuRF n=16 261	P value
Heart rate (beats per minute), mean (SD)		76.0 (16.7)	76.9 (17.2)	75.8 (16.6)	0.009
Systolic blood pressure (mm Hg), mean (SD)		127.6 (25.2)	124.8 (23.9)	128.1 (25.4)	<0.001
Diastolic blood pressure (mm Hg), mean (SD)		74.2 (15.1)	73.6 (14.8)	74.3 (15.1)	0.053
Cardiogenic shock, n (%)		862 (4.5%)	141 (5.2%)	721 (4.4%)	0.087
Out-of-hospital cardiac arrest, n (%)		819 (4.3%)	179 (6.6%)	640 (3.9%)	<0.001
ACS presentation, n (%)	STEMI	9870 (52.0%)	1611 (59.1%)	8259 (50.8%)	<0.001
	NSTEMI	7676 (40.4%)	975 (35.8%)	6701 (41.2%)	
	UA	1442 (7.6%)	141 (5.2%)	1301 (8.0%)	
Killip Class, n (%)	1	14 100 (74.3%)	2088 (76.6%)	12 012 (73.9%)	0.003
	≥2	4888 (25.7%)	639 (23.4%)	4249 (26.1%)	
Ejection fraction (%), mean (SD)		51.7 (10.0)	50.8 (10.2)	51.8 (10.0)	<0.001
Ejection fraction (%)	>45	13 455 (76.2%)	1881 (73.3%)	11 574 (76.7%)	<0.001
	30–45	3981 (22.5%)	642 (25.0%)	3339 (22.1%)	
	<30	226 (1.3%)	42 (1.6%)	184 (1.2%)	
NYHA Class, n (%)	I	10 862 (69.5%)	1759 (75.7%)	9103 (68.4%)	<0.001
	II	1578 (10.1%)	196 (8.4%)	1382 (10.4%)	
	III	774 (5.0%)	72 (3.1%)	702 (5.3%)	
	IV	2418 (15.5%)	296 (12.7%)	2122 (15.9%)	
Extent of coronary disease, n (%)	Single vessel	9847 (52.0%)	1556 (57.1%)	8291 (51.1%)	<0.001
	Multi vessel	9102 (48.0%)	1169 (42.9%)	7933 (48.9%)	
Target lesion, n (%)	LAD	7653 (40.3%)	1259 (46.2%)	6394 (39.3%)	<0.001
	Circumflex	2319 (12.2%)	268 (9.8%)	2051 (12.6%)	
	RCA	6555 (34.5%)	854 (31.3%)	5701 (35.1%)	
Ostial lesion present, n (%)		1421 (7.5%)	245 (9.0%)	1176 (7.2%)	0.001
TIMI flow (pre), n (%)	0	6895 (36.4%)	1175 (43.2%)	5720 (35.2%)	<0.001
	1	937 (4.9%)	137 (5.0%)	800 (4.9%)	
	2	2095 (11.1%)	317 (11.6%)	1778 (11.0%)	
	3	9032 (47.6%)	1094 (40.2%)	7938 (48.9%)	
TIMI flow (post), n (%)	0	386 (2.0%)	54 (2.0%)	332 (2.0%)	0.32
	1	87 (0.5%)	9 (0.3%)	78 (0.5%)	
	2	400 (2.1%)	68 (2.5%)	332 (2.0%)	
	3	18 108 (95.4%)	2593 (95.2%)	15 515 (95.4%)	
No reflow, n (%)	No	17 637 (95.9%)	2533 (94.9%)	15 104 (96.1%)	0.012
	Transient	600 (3.3%)	111 (4.2%)	489 (3.1%)	
	Persistent	151 (0.8%)	26 (1.0%)	125 (0.8%)	
Percutaneous entry location, n (%)	Brachial or radial	7114 (37.5%)	1061 (38.9%)	6053 (37.2%)	0.093
	Femoral	11 874 (62.5%)	1666 (61.1%)	10 208 (62.8%)	
Intra-aortic balloon pump required, n (%)		447 (4.7%)	89 (7.2%)	358 (4.3%)	<0.001
Device deployed, n (%)	Balloon only	846 (4.5%)	89 (3.3%)	757 (4.7%)	0.001
	BMS	6804 (35.8%)	934 (34.3%)	5870 (36.1%)	0.062
	DES	11 435 (60.2%)	1690 (62.0%)	9745 (59.9%)	0.044

ACS, acute coronary syndrome; BMS, bare metal stent; DES, drug-eluting stent; LAD, left anterior descending; NSTEMI, non-ST-elevation myocardial infarction; NYHA, New York Heart Association; RCA, right coronary artery; SD, standard deviation; STEMI, ST-elevation myocardial infarction; TIMI, Thrombolysis in Myocardial Infarction; UA, unstable angina.

### Patient outcomes

Almost 1% more SMuRF-less patients experienced postprocedural cardiogenic shock (p=0.019) and just over 1% more experienced arrhythmia (p=0.029) (table 3). No other differences with in-hospital complications between the two groups were found. Median (IQR) length of stay for all patients was 4 (3, 5) days.

**Table 3:** In-hospital outcomes of the cohort stratified by the presence or absence of standard modifiable risk factors (SMuRFs)

In-hospital outcome	Total n=18 988	SMuRF less n=2727	≥1 SMuRF n=16 261	P value
Emergency PCI, n (%)	129 (0.7%)	20 (0.7%)	109 (0.7%)	0.71
Unplanned CABG, n (%)	178 (0.9%)	22 (0.8%)	156 (1.0%)	0.45
Cardiogenic shock, n (%)	707 (3.7%)	123 (4.5%)	584 (3.6%)	0.019
Arrhythmia, n (%)	1910 (10.1%)	306 (11.2%)	1604 (9.9%)	0.029
Stroke, n (%)				0.17
No	18 908 (99.6%)	2718 (99.7%)	16 190 (99.6%)	
Haemorrhagic	20 (0.1%)	0 (0.0%)	20 (0.1%)	
Ischaemic	56 (0.3%)	9 (0.3%)	47 (0.3%)	
New renal impairment, n (%)	505 (2.7%)	74 (2.7%)	431 (2.7%)	0.85
Heart failure, n (%)	914 (4.8%)	130 (4.8%)	784 (4.8%)	0.90
Length of stay (days), median (IQR)	4.0 (3.0, 5.0)	4.0 (3.0, 5.0)	4.0 (3.0, 5.0)	<0.001
New or recurrent MI, n (%)	165 (0.9%)	25 (0.9%)	140 (0.9%)	0.77
Bleed, n (%)				0.92
No	18 528 (97.6%)	2662 (97.6%)	15 866 (97.6%)	
Yes	458 (2.4%)	65 (2.4%)	393 (2.4%)	
Bleed requiring transfusion, n (%)	284 (1.5%)	43 (1.6%)	241 (1.5%)	0.75
MACCE, n (%)	920 (4.8%)	139 (5.1%)	781 (4.8%)	0.51
In-hospital mortality, n (%)	510 (2.7%)	83 (3.0%)	427 (2.6%)	0.21

CABG, coronary artery bypass graft surgery; IQR, interquartile range; MACCE, major adverse cardiac and cerebrovascular events; MI, myocardial infarction; PCI, percutaneous coronary intervention.

At 30-day postprocedure, no differences between patients with and without SMuRFs were identified with regards to mortality, AMI, TVR, MACE or MACCE (table 4). Even after controlling for potential confounders, no differences between the groups were found in 30-day outcomes (online supplemental tables 1–5). Some differences were, however, identified in the prescription of guideline-directed post-PCI medications (table 5). SMuRF-less patients were less likely to be prescribed ACE inhibitors or angiotensin receptor blockers (ARBs; 79.4% vs 83.8%) compared with their counterparts with ≥1 SMuRF.

**Table 4:** 30-day outcomes of the cohort stratified by the presence or absence of standard modifiable risk factors (SMuRFs)

30-day outcome	Total n=18 988	SMuRF less n=2727	≥1 SMuRF n=16 261	P value
All-cause mortality, n (%)	615 (3.3%)	97 (3.6%)	518 (3.2%)	0.31
Acute myocardial infarction, n (%)	270 (1.4%)	34 (1.3%)	236 (1.5%)	0.40
Target vessel revascularisation, n (%)	457 (2.4%)	60 (2.2%)	397 (2.4%)	0.45
MACE, n (%)	1167 (6.2%)	166 (6.1%)	1001 (6.2%)	0.89
MACCE, n (%)	1247 (6.6%)	175 (6.4%)	1072 (6.6%)	0.73

MACCE, major adverse cardiac and cerebrovascular events; MACE, major adverse cardiovascular events.

**Table 5:** 30-day prescription of guideline-directed post-PCI medications stratified by the presence or absence of standard modifiable risk factors (SMuRFs)

Prescribed at 30 days	Total n=18 988	SMuRF-less n=2727	≥1 SMuRF n=16 261	P value
Aspirin, n (%)	16 966 (97.3%)	2454 (97.3%)	14 512 (97.3%)	0.99
Clopidogrel/prasugrel/ticagrelor, n (%)	16 865 (96.8%)	2438 (96.6%)	14 427 (96.8%)	0.66
Clopidogrel, n (%)	9213 (52.9%)	1230 (48.8%)	7983 (53.6%)	<0.001
Prasugrel, n (%)	1267 (9.4%)	193 (9.6%)	1074 (9.4%)	0.72
Ticagrelor, n (%)	6396 (56.5%)	1019 (59.6%)	5377 (55.9%)	0.005
Statin/fibrate/ezetimibe, n (%)	16 867 (97.0%)	2436 (96.9%)	14 431 (97.0%)	0.68
Beta blocker, n (%)	14 509 (83.6%)	2094 (83.5%)	12 415 (83.7%)	0.83
ACE inhibitor/ARB, n (%)	14 427 (83.1%)	1993 (79.4%)	12 434 (83.8%)	<0.001
Warfarin, n (%)	1084 (6.2%)	189 (7.5%)	895 (6.0%)	0.004
Spironolactone/eplerenone, n (%)	820 (5.1%)	127 (5.5%)	693 (5.1%)	0.37

ACE, angiotensin-converting enzyme; ARB, angiotensin receptor blocker; PCI, percutaneous coronary intervention.

**Figure 1:**
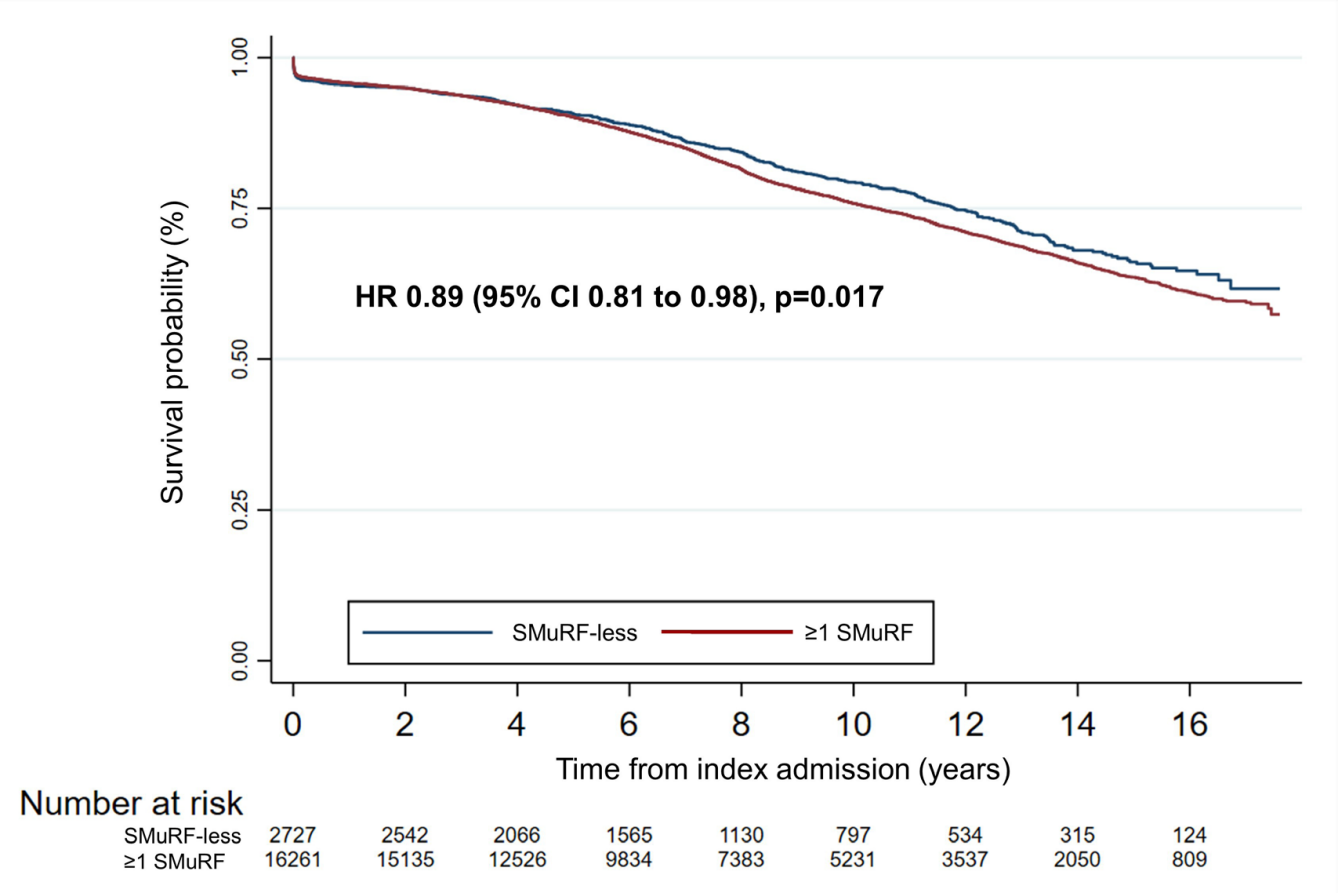
Unadjusted Cox regression analysis of long-term mortality by SMuRF status. SMuRF, standard modifiable risk factor.

**Figure 2:**
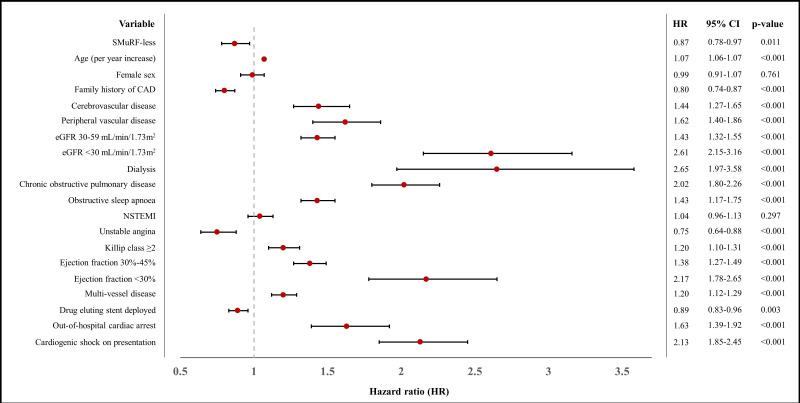
Forest plot representing results of the adjusted Cox regression analysis of long-term mortality within the cohort. ACS, acute coronary syndrome; CAD, coronary artery disease; eGFR, estimated glomerular filtration rate; NSTEMI, non-ST-elevation myocardial infarction; SMuRF, standard modifiable risk factor; STEMI, ST-elevation myocardial infarction; UA, unstable angina.

Mortality at 12 months did not differ between the two groups (table 6). However, mortality at any time during follow-up occurred less frequently in SMuRF-less patients (18.1% vs 21.4% in those with ≥1 SMuRF, p<0.001). For SMuRF-less patients who died, the median (IQR) time to mortality was 6 months shorter than in those with ≥1 SMuRF (6.9 (4.1–10.9) years vs 7.4 (4.2–11.4) years, p<0.001). Unadjusted Cox proportional hazards regression analysis revealed that SMuRF-less patients had an 11% lower hazard of death during follow-up and, therefore, improved survival than patients with SMuRFs (HR 0.89 (95% CI 0.81 to 0.98), p=0.017) (figure 1). On adjusted Cox regression analysis, SMuRF-less patients further demonstrated a decreased rate of mortality by 13% compared with those with SMuRFs (HR 0.87 (95% CI 0.78 to 0.97), p=0.011) (figure 2).

**Table 6:** Long-term mortality stratified by the presence or absence of standard modifiable risk factors (SMuRFs)

	Total n=18 988	SMuRF-less n=2727	≥1 SMuRF n=16 261	P value
12-month mortality, n (%)	934 (4.9%)	143 (5.2%)	791 (4.9%)	0.400
Mortality at any time during follow-up, n (%)	3975 (20.9%)	494 (18.1%)	3481 (21.4%)	<0.001
Time to mortality (years), median (IQR)	7.3 (4.2–11.3)	6.9 (4.1–10.9)	7.4 (4.2–11.4)	<0.001

IQR, interquartile range.

### Stratified analyses

We found no significant differences in 30-day mortality between SMuRF-less patients and those with SMuRFs in any subgroups including females only, males only, aged ≤65 years, aged >65 years, STEMI and NSTEMI (online supplemental table 6).

## Discussion

In this study of patients undergoing PCI for ACS where we compared characteristics and outcomes of patients with and without SMuRFs, we made three key findings. First, being SMuRF-less was not uncommon in our cohort, with 1 in 7 patients (14%) fulfilling the criteria defining this patient group. In addition, we identified a significant increase in the proportion of SMuRF-less patients over time. Second, although similar in age, SMuRF-less patients demonstrated a less clinically complex profile at baseline with lower rates of comorbidities commonly associated with poorer outcomes in ACS. SMuRF-less patients were, however, more likely to present with a catastrophic cardiac event, namely OHCA and obstruction of the LAD and were more likely to experience postprocedural cardiogenic shock and arrhythmia. Lastly, there was no difference in short-term outcomes between those with or without SMuRFs. However, being SMuRF-less was independently associated with improved long-term survival.

Many studies report similar characteristics and in-hospital events, but a differing proportion of patients classified as SMuRF-less. In the ARIC Study, just 3.6% of patients with AMI hospitalised between 2000 and 2014 were SMuRF-less.^13^ In a meta-analysis including a total of 15 studies involving approximately 1.3 million patients with ACS, 11.6% were SMuRF-less.^14^ Vernon *et al* (2019) used the Cooperative National Registry of Acute Coronary care, Guideline Adherence, and Clinical Events (CONCORDANCE) Registry, and the Australian GRACE and found that in Australia, the proportion of patients with SMuRF-less STEMI had risen significantly from 14% in 1999 to 23% in 2017.^7^ We also found that the proportion of SMuRF-less patients increased significantly over time, although not to the same magnitude. This pattern may be the result of improved identification and management of modifiable risk factors in primary care (and therefore, a decrease in events in patients with SMuRFs) or may indicate that there are additional factors yet to be identified causing SMuRF-less patients to experience acute cardiac events at increasing rates.

Our finding that SMuRF-less patients more often experience critical ACS with significantly increased proportions of catastrophic cardiac features both on presentation and in-hospital have also been demonstrated by others.^8 13 15–19^ The finding is consistent across many studies and regions—approximately 1.5–3 times the number of SMuRF-less patients than patients with SMuRFs present with cardiac arrest or cardiogenic shock and/or experience these events in hospital.^13–17 20–22^ ACS complicated by OHCA or cardiogenic shock leads to larger infarct size, prolonged organ hypoperfusion and more severe myocardial injury. As a result, prognosis in these patients is expected to be poorer, and some groups have observed this while we and others have not.

In mediation analysis conducted by Kelly *et al* (2023), increased 30-day mortality in patients with SMuRF-less STEMI was partially mediated by higher rates of cardiac arrest and LAD territory events.^17^ The consistent finding by us and others of an increased presence of high-risk coronary lesions in SMuRF-less patients (eg, TIMI flow grade 0/1 pre-PCI or LAD involvement) is significant and may be reflective of the presence of a vulnerable plaque and higher lipid core in SMuRF-less patients.^15 23^ In a pooled analysis of individual patient-level data from 10 randomised trials that enrolled patients with STEMI undergoing PCI, adjustment of models for TIMI flow 0/1 pre-PCI resulted in no difference in 30-day or 12-month outcomes between SMuRF-less patients and those with ≥1 SMuRF.^23^ A study of >86 500 patients with AMI in a multiethnic AMI registry found that in-hospital mortality and 12-month cardiovascular mortality were significantly higher in patients with SMuRF-less STEMI after adjustment for age, creatinine and haemoglobin.^20^ However, the difference was no longer significant after additional adjustment for anterior infarction, cardiopulmonary resuscitation (ie, cardiac arrest) and Killip Class,^20^ all of which we identified as significantly different between the groups.

Our findings are similar to those of Anderson *et al*.^24^ In a group of patients with STEMI who attended cardiac catheterisation laboratories in the USA, 26.2% were SMuRF-less. SMuRF-less patients were younger, more frequently male and had fewer comorbidities, as in our study. No differences in short-term outcomes were identified and long-term outcomes were more frequent in patients with ≥1 SMuRF, again reflective of our findings and that SMuRF-less patients have fewer comorbidities and life-limiting risk factors (eg, diabetes and hypertension). Anderson *et al* also looked at patients with NSTEMI of whom 17.8% were SMuRF-less.^25^ Both short-term and long-term outcomes favoured SMuRF-less patients. Others have also shown no difference in postdischarge short-term and longer-term mortality between the two groups.^16 20^ Contradicting these findings, the SWEDEHEART National Registry (including all patients admitted to cardiac care units in Sweden with MI^26 27^) demonstrated poorer outcomes in SMuRF-less patients. Patients with SMuRF-less STEMI experienced a 47% higher 30-day mortality compared with their SMuRF counterparts.^8^ This early mortality, however, was attenuated over the longer term with SMuRF-less patients experiencing lower adjusted all-cause mortality at 5 years.^8^

Factors within broader systems of care may contribute to differences in outcomes and need to be explored further. Favourable outcomes in AMI are time dependent and, therefore, key time metrics should be considered such as symptom-to-balloon time, and door-to-balloon time for those undergoing revascularisation. Some groups have compared these metrics between SMuRF-less patients and those with ≥1 SMuRF, with studies indicating longer times reflecting poorer outcomes and those with no difference between the groups showing similar or better outcomes in SMuRF-less patients.^19 22^ Prehospital management should also be considered as should postdischarge pharmacological prescribing (as we and others have done^19 28^), medication adherence and lifestyle changes, all of which influence outcomes. Some have found that suboptimal provision of multiple guideline-directed therapies (beta-blockers, ACE/ARB, statins) partially mediated higher mortality rates in SMuRF-less patients.^8 21 22 29 30^

Despite the similarities with some groups and differences with others regarding our findings, all highlight the importance of this subgroup of patients with ACS who require intervention for a disease process not predicted by SMuRFs alone. Improved phenotyping of patients with ACS involving the development and use of biomarkers and non-invasive imaging is crucial for the identification of risk and prevention of atherosclerotic development, progression and catastrophic presentation in this patient group.

### Limitations

There are, of course, limitations of this study. First, given that the MIG registry doesn’t contain data from all PCI-capable hospitals, there is a risk of selection bias and generalisability of findings may be restricted. In addition, SMuRF-less status varies by country and presented data may not necessarily be generalisable to populations outside of Australia. However, MIG is multicentred and contains comprehensive data on many procedures collected over nearly two decades. Second, outcomes beyond 30 days are limited to all-cause mortality, with data relating to longer term cardiovascular-specific outcomes and hospital readmissions not available in the dataset. Third, while we aimed to include a broad array of potential confounders in multivariable models, residual confounding, or unmeasured confounding due to variables not being available in the MIG dataset cannot be excluded. For example, as data on time intervals (eg, symptom onset to balloon time) was not available in the MIG dataset, we were unable to identify whether SMuRF-less individuals delayed seeking treatment as a result of not expecting to be at risk for ACS. In addition, although standard and rigorous methodology was used to collect data within the MIG registry, there is a possibility that SMuRF-less patients may have been misclassified due to missing data on risk factors present at baseline. This is particularly true for patients who presented in cardiac arrest or cardiogenic shock where it was likely not possible to obtain data on current risk factors. Similarly, patients being SMuRF-less may have been a misclassification simply because they never had tests performed and any risk factors were yet to be identified. It is, therefore, likely that a proportion of SMuRF-less patients did have risk factors for ACS. Finally, our cohort consisted of patients with ACS undergoing PCI and findings may not be applicable to patients without revascularisation.

## Conclusion

In our study cohort, the proportion of patients with SMuRF-less ACS significantly increased over time. SMuRF-less patients were more likely to present with OHCA and obstruction of the LAD and were more likely to experience postprocedural cardiogenic shock and arrhythmia. For these patients, 30-day outcomes were similar, and long-term outcomes were more favourable than in those with SMuRFs. Further research is needed to identify high-risk SMuRF-less individuals.

## Data Availability

Data are available upon reasonable request. The dataset used and/or analysed during the current study is available from the MIG investigators upon reasonable request.
