# Supplementary material for: Prevalence and outcomes of patients with SMuRF-less acute coronary syndrome undergoing percutaneous coronary intervention
Source: Open Heart. 2024 Jun 5;11(1):e002733. doi: 10.1136/openhrt-2024-002733 (PMC11163639; doi:10.1136/openhrt-2024-002733)
Supplement: Supplementary data [file openhrt-2024-002733supp001.pdf]

Supplemental Material

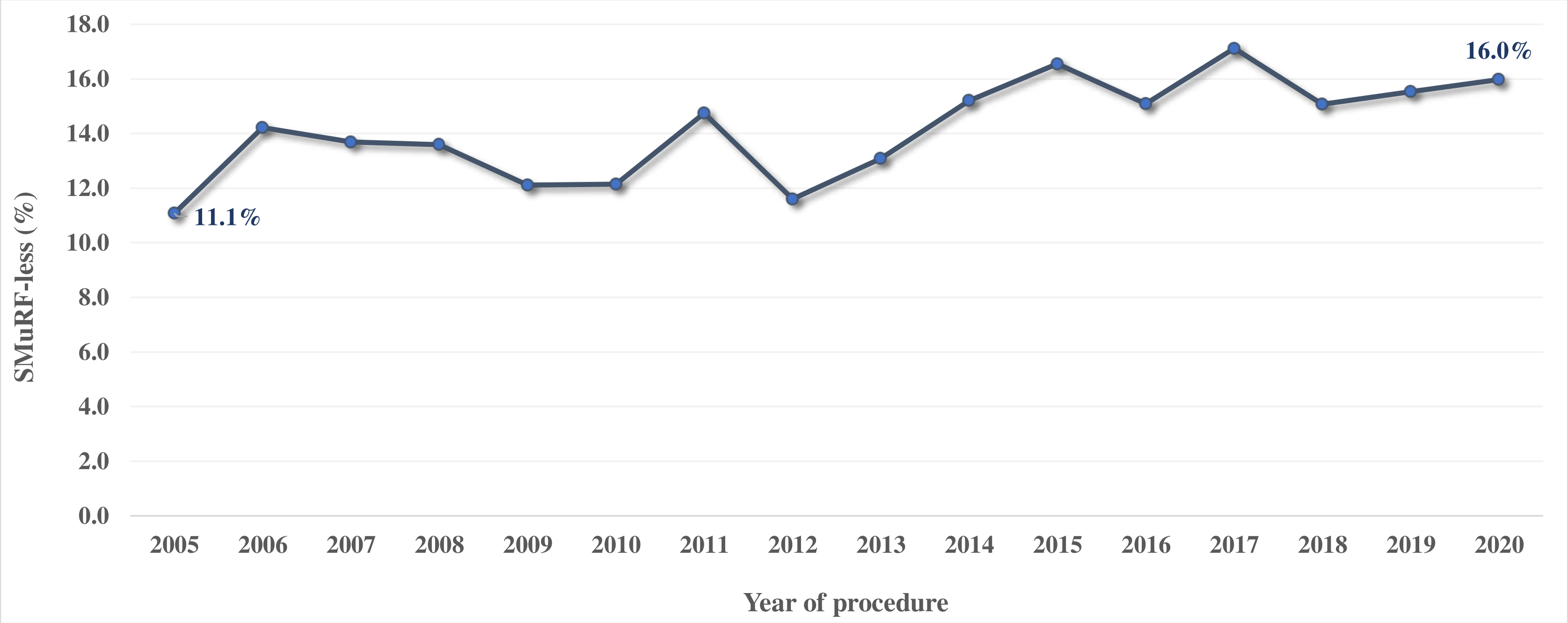

Supplementary Figure 1: Change over time in the proportion of SMuRF-less patients during the study period

SMuRF = standard modifiable risk factor

Ball J, *et al. Open Heart* 2024; 11:e002733. doi: 10.1136/openhrt-2024-002733

Supplementary Table 1: Adjusted odds ratios of 30-day mortality within the cohort

Variable	Category	Odds ratio	95% CI	p-value
SMuRF-less		0.91	0.63-1.31	0.608
Age (per year increase)		1.04	1.03-1.05	<0.001
Female sex		0.90	0.67-1.21	0.498
Cerebrovascular disease		1.25	0.73-2.12	0.415
Peripheral vascular disease		1.37	0.80-2.33	0.247
Family history of CAD		0.65	0.47-0.90	0.010
eGFR (ml/min/1.73m^2)	≥60	*Reference*		
	30 to <60	2.17	1.65-2.87	<0.001
	<30	2.72	1.48-5.01	0.001
Dialysis		1.95	0.66-5.75	0.228
Chronic obstructive pulmonary disease		1.75	1.12-2.74	0.014
Obstructive sleep apnoea		0.93	0.39-2.23	0.877
ACS presentation	STEMI	*Reference*		
	NSTEMI	0.80	0.56-1.15	0.222
	UA	0.45	0.19-1.11	0.082
Killip class	1	*Reference*		
	≥2	1.65	1.21-2.27	0.002
Ejection fraction (%)	>45	*Reference*		
	30-45	2.03	1.53-2.69	<0.001
	<30	3.74	2.24-6.26	<0.001
Extent of coronary disease	Single vessel	*Reference*		
	Multi vessel	1.13	0.87-1.48	0.358
TIMI flow pre-PCI	0	*Reference*		
	1	0.98	0.57-1.68	0.928
	2	0.76	0.48-1.22	0.257

Ball J, *et al. Open Heart* 2024; 11:e002733. doi: 10.1136/openhrt-2024-002733

	3	0.82	0.58-1.16	0.258
Drug eluting stent deployed		1.07	0.82-1.39	0.629
Cardiogenic shock on presentation		2.30	1.58-3.34	<0.001
Out-of-hospital cardiac arrest on presentation		4.52	3.24-6.32	<0.001
Cardiogenic shock post-procedure		7.71	5.44-10.92	<0.001
Arrhythmia post-procedure		1.87	1.40-2.51	<0.001

SMuRF = standard modifiable risk factor; CAD = coronary artery disease; eGFR = estimated glomerular filtration rate; ACS = acute coronary syndrome; STEMI = ST-elevation myocardial infarction; NSTEMI = non-ST-elevation myocardial infarction; UA = unstable angina; PCI = percutaneous coronary intervention

Ball J, *et al. Open Heart* 2024; 11:e002733. doi: 10.1136/openhrt-2024-002733

Supplementary Table 2: Adjusted odds ratios of 30-day AMI within the cohort

Variable	Category	Odds ratio	95% CI	p-value
SMuRF-less		0.91	0.61-1.36	0.653
Age (per year increase)		1.00	0.99-1.01	0.801
Female sex		1.34	1.00-1.81	0.052
Cerebrovascular disease		1.12	0.60-2.10	0.723
Peripheral vascular disease		0.57	0.24-1.35	0.201
Family history of CAD		0.82	0.61-1.11	0.203
eGFR (ml/min/1.73m^2)	≥60	*Reference*		
	30 to <60	0.84	0.58-1.22	0.361
	<30	1.19	0.51-2.77	0.686
Dialysis		3.47	1.11-10.81	0.032
Chronic obstructive pulmonary disease		1.34	0.79-2.27	0.282
Obstructive sleep apnoea		0.60	0.22-1.62	0.309
ACS presentation	STEMI	*Reference*		
	NSTEMI	0.92	0.67-1.28	0.635
	UA	0.84	0.45-1.55	0.569
Killip class	1	*Reference*		
	≥2	1.47	1.05-2.06	0.026
Ejection fraction (%)	>45	*Reference*		
	30-45	1.53	1.13-2.08	0.006
	<30	0.78	0.27-2.23	0.640
Extent of coronary disease	Single vessel	*Reference*		
	Multi vessel	1.51	1.14-1.98	0.004
TIMI flow pre-PCI	0	*Reference*		
	1	1.22	0.67-2.22	0.522
	2	0.92	0.56-1.53	0.760

	3	1.28	0.91-1.80	0.155
Drug eluting stent deployed		0.80	0.61-1.05	0.115
Cardiogenic shock on presentation		0.79	0.41-1.49	0.463
Out-of-hospital cardiac arrest on presentation		0.87	0.47-1.63	0.674
Cardiogenic shock post-procedure		3.02	1.67-5.47	<0.001
Arrhythmia post-procedure		1.68	1.14-2.48	0.009

SMuRF = standard modifiable risk factor; CAD = coronary artery disease; eGFR = estimated glomerular filtration rate; ACS = acute coronary syndrome; STEMI = ST-elevation myocardial infarction; NSTEMI = non-ST-elevation myocardial infarction; UA = unstable angina; PCI = percutaneous coronary intervention

Ball J, *et al. Open Heart* 2024; 11:e002733. doi: 10.1136/openhrt-2024-002733

Supplementary Table 3: Adjusted odds ratios of 30-day target vessel revascularisation within the cohort

Variable	Category	Odds ratio	95% CI	p-value
SMuRF-less		0.89	0.65-1.22	0.454
Age (per year increase)		0.99	0.98-1.00	0.133
Female sex		0.97	0.75-1.26	0.840
Cerebrovascular disease		0.66	0.34-1.25	0.202
Peripheral vascular disease		0.76	0.38-1.53	0.448
Family history of CAD		1.16	0.92-1.45	0.209
eGFR (ml/min/1.73m^2)	≥60	*Reference*		
	30 to <60	1.16	0.88-1.53	0.290
	<30	0.29	0.09-0.91	0.034
Dialysis		4.16	0.94-18.45	0.061
Chronic obstructive pulmonary disease		0.76	0.43-1.32	0.325
Obstructive sleep apnoea		1.13	0.61-2.10	0.688
ACS presentation	STEMI	*Reference*		
	NSTEMI	1.01	0.78-1.32	0.918
	UA	0.86	0.48-1.54	0.616
Killip class	1	*Reference*		
	≥2	1.14	0.87-1.51	0.344
Ejection fraction (%)	>45	*Reference*		
	30-45	1.17	0.92-1.50	0.200
	<30	0.67	0.31-1.46	0.312
Extent of coronary disease	Single vessel	*Reference*		
	Multi vessel	2.75	2.17-3.48	<0.001
TIMI flow pre-PCI	0	*Reference*		
	1	0.85	0.52-1.39	0.519

Ball J, *et al. Open Heart* 2024; 11:e002733. doi: 10.1136/openhrt-2024-002733

	2	0.84	0.58-1.20	0.328
	3	0.62	0.47-0.81	0.001
Drug eluting stent deployed		0.42	0.34-0.52	<0.001
Cardiogenic shock on presentation		0.95	0.59-1.52	0.823
Out-of-hospital cardiac arrest on presentation		0.93	0.59-1.45	0.744
Cardiogenic shock post-procedure		3.07	1.97-4.79	<0.001
Arrhythmia post-procedure		2.01	1.50-2.69	<0.001

SMuRF = standard modifiable risk factor; CAD = coronary artery disease; eGFR = estimated glomerular filtration rate; ACS = acute coronary syndrome; STEMI = ST-elevation myocardial infarction; NSTEMI = non-ST-elevation myocardial infarction; UA = unstable angina; PCI = percutaneous coronary intervention

Ball J, *et al. Open Heart* 2024; 11:e002733. doi: 10.1136/openhrt-2024-002733

Supplementary Table 4: Adjusted odds ratios of 30-day MACE within the cohort

Variable	Category	Odds ratio	95% CI	p-value
SMuRF-less		0.88	0.70-1.12	0.302
Age (per year increase)		1.01	1.00-1.02	0.013
Female sex		1.01	0.84-1.22	0.907
Cerebrovascular disease		1.07	0.74-1.53	0.729
Peripheral vascular disease		0.87	0.58-1.32	0.524
Family history of CAD		0.95	0.80-1.13	0.577
eGFR (ml/min/1.73m^2)	≥60	*Reference*		
	30 to <60	1.43	1.19-1.73	<0.001
	<30	1.39	0.84-2.30	0.206
Dialysis		2.58	1.18-5.67	0.018
Chronic obstructive pulmonary disease		1.32	0.97-1.81	0.081
Obstructive sleep apnoea		0.92	0.56-1.50	0.739
ACS presentation	STEMI	*Reference*		
	NSTEMI	1.02	0.84-1.24	0.839
	UA	0.73	0.48-1.11	0.138
Killip class	1	*Reference*		
	≥2	1.42	1.17-1.72	<0.001
Ejection fraction (%)	>45	*Reference*		
	30-45	1.56	1.31-1.86	<0.001
	<30	1.95	1.27-3.01	0.002
Extent of coronary disease	Single vessel	*Reference*		
	Multi vessel	1.84	1.56-2.17	<0.001
TIMI flow pre-PCI	0	*Reference*		
	1	0.94	0.66-1.34	0.745
	2	0.84	0.64-1.10	0.206

Ball J, *et al. Open Heart* 2024; 11:e002733. doi: 10.1136/openhrt-2024-002733

	3	0.78	0.63-0.95	0.014
Drug eluting stent deployed		0.60	0.51-0.70	<0.001
Cardiogenic shock on presentation		1.61	1.20-2.17	0.002
Out-of-hospital cardiac arrest on presentation		2.29	1.75-2.99	<0.001
Cardiogenic shock post-procedure		5.81	4.40-7.68	<0.001
Arrhythmia post-procedure		1.82	1.49-2.24	<0.001

SMuRF = standard modifiable risk factor; CAD = coronary artery disease; eGFR = estimated glomerular filtration rate; ACS = acute coronary syndrome; STEMI = ST-elevation myocardial infarction; NSTEMI = non-ST-elevation myocardial infarction; UA = unstable angina; PCI = percutaneous coronary intervention

Supplementary Table 5: Adjusted odds ratios of 30-day MACCE within the cohort

Variable	Category	Odds ratio	95% CI	p-value
SMuRF-less		0.88	0.70-1.09	0.242
Age (per year increase)		1.01	1.00-1.02	0.003
Female sex		1.03	0.86-1.23	0.764
Cerebrovascular disease		1.03	0.72-1.46	0.891
Peripheral vascular disease		0.79	0.52-1.19	0.257
Family history of CAD		0.92	0.78-1.09	0.359
eGFR (ml/min/1.73m^2)	≥60	*Reference*		
	30 to <60	1.43	1.20-1.72	<0.001
	<30	1.43	0.88-2.35	0.150
Dialysis		2.59	1.21-5.54	0.015
Chronic obstructive pulmonary disease		1.35	1.00-1.82	0.054
Obstructive sleep apnoea		0.94	0.59-1.50	0.804
ACS presentation	STEMI	*Reference*		
	NSTEMI	0.98	0.81-1.89	0.866
	UA	0.71	0.47-1.07	0.098
Killip class	1	*Reference*		
	≥2	1.34	1.11-1.62	0.003
Ejection fraction (%)	>45	*Reference<0.001*		
	30-45	1.55	1.31-1.84	<0.001
	<30	1.90	1.24-2.90	0.003
Extent of coronary disease	Single vessel	*Reference*		
	Multi vessel	1.75	1.49-2.05	<0.001
TIMI flow pre-PCI	0	*Reference*		
	1	0.96	0.68-1.34	0.796
	2	0.82	0.63-1.07	0.147

	3	0.79	0.65-0.96	0.017
Drug eluting stent deployed		0.62	0.53-0.72	<0.001
Cardiogenic shock on presentation		1.74	1.30-2.32	<0.001
Out-of-hospital cardiac arrest on presentation		2.05	1.57-2.67	<0.001
Cardiogenic shock post-procedure		5.33	4.05-7.01	<0.001
Arrhythmia post-procedure		1.95	1.60-2.37	<0.001

SMuRF = standard modifiable risk factor; CAD = coronary artery disease; eGFR = estimated glomerular filtration rate; ACS = acute coronary syndrome; STEMI = ST-elevation myocardial infarction; NSTEMI = non-ST-elevation myocardial infarction; UA = unstable angina; PCI = percutaneous coronary intervention

Ball J, *et al. Open Heart* 2024; 11:e002733. doi: 10.1136/openhrt-2024-002733

Supplementary Table 6: 30-day mortality in subgroups stratified by the presence or absence of SMuRFs

Patient subgroup	Total deaths	SMuRF-less	≥1 SMuRF	p-value
Females, n (%)<br>(n=4,671)	189 (4.0%)	19 (3.5%)	170 (4.1%)	0.508
Males, n (%)<br>(n=14,316)	426 (3.0%)	78 (3.6%)	348 (2.9%)	0.077
Aged ≤65 years, n (%)<br>(n=11,207)	236 (2.1%)	36 (2.4%)	200 (2.1%)	0.438
Aged >65 years, n (%)<br>(n=7,781)	379 (4.9%)	61 (5.1%)	318 (4.8%)	0.759
STEMI, n (%)<br>(n=9,870)	522 (5.3%)	85 (5.3%)	437 (5.3%)	0.980
NSTEMI, n (%)<br>(n=7,676)	87 (1.1%)	12 (1.2%)	75 (1.1%)	0.759
